# PER2 mediates CREB-dependent light induction of the clock gene *Per1*

**DOI:** 10.1038/s41598-021-01178-6

**Published:** 2021-11-05

**Authors:** Andrea Brenna, Jürgen A. Ripperger, Gabriella Saro, Dominique A. Glauser, Zhihong Yang, Urs Albrecht

**Affiliations:** 1 Department of Biology, Faculty of Science and Medicine, University of Fribourg, Fribourg, Switzerland; 2 Laboratory of Cardiovascular and Aging Research, Department of Endocrinology, Metabolism, Cardiovascular System, Faculty of Science and Medicine, University of Fribourg, Fribourg, Switzerland

**Keywords:** Cell biology, Circadian rhythms

## Abstract

Light affects many physiological processes in mammals such as entrainment of the circadian clock, regulation of mood, and relaxation of blood vessels. At the molecular level, a stimulus such as light initiates a cascade of kinases that phosphorylate CREB at various sites, including serine 133 (S133). This modification leads CREB to recruit the co-factor CRCT1 and the histone acetyltransferase CBP to stimulate the transcription of genes containing a CRE element in their promoters, such as *Period 1* (*Per1*). However, the details of this pathway are poorly understood. Here we provide evidence that PER2 acts as a co-factor of CREB to facilitate the formation of a transactivation complex on the CRE element of the *Per1* gene regulatory region in response to light or forskolin. Using *in vitro* and *in vivo* approaches, we show that PER2 modulates the interaction between CREB and its co-regulator CRTC1 to support complex formation only after a light or forskolin stimulus. Furthermore, the absence of PER2 abolished the interaction between the histone acetyltransferase CBP and CREB. This process was accompanied by a reduction of histone H3 acetylation and decreased recruitment of RNA Pol II to the *Per1* gene. Collectively, our data show that PER2 supports the stimulus-dependent induction of the *Per1* gene via modulation of the CREB/CRTC1/CBP complex.

## Introduction

Light perception is one of the most important mechanisms that provoke biological responses in organisms, from resetting the circadian clock to cell division, metabolism, and redox state regulation^1–5^. In mammals, light can entrain the circadian clock, regulate mood behaviors, and blood vessel relaxation^6–8^. Light is perceived by the retina by intrinsically photosensitive retinal ganglion cells (ipRGCs) and transduced via the retinohypothalamic tract (RHT) to many different brain regions. For instance, in the suprachiasmatic nuclei (SCN) located above the optic chiasm in the ventral part of the hypothalamus, the signal is responsible for the release of neurotransmitters such as glutamate and PACAP^9^. Light-evoked signals provoke Ca^2+^ influx in SCN neurons and activate a phosphorylation cascade, including protein kinase A (PKA), protein kinase G (PKG), Ca^2+^/calmodulin-dependent protein kinase (CaMK), and mitogen-activated protein kinases (MAPK) also known as extracellular-signal-regulated kinases (ERK). This cascade finally promotes phosphorylation of cAMP response element-binding protein (CREB) at serine 133 and 142^10–12^. CREB dimers recognize a specific motif of an 8-base pair palindromic sequence (TGACGTCA) called cAMP response element (CRE) present on regulatory regions of target genes such as *Per1*^13,14^. In neurons, cAMP-regulated transcriptional co-activator 1 (CRTC1) is required for efficient induction of CREB target genes during neuronal activity^15,16^. Upon stimulation of L-type voltage-gated calcium channels, CRTC1 is dephosphorylated by calcineurin. Subsequently, it translocates into the nucleus, where it interacts via its N-terminal CREB-binding domain (CBD) with CREB^17^. Finally, the histone acetyltransferase CREB binding protein (CBP) binds to phospho-Ser133 CREB (pS133-CREB)^18^. The subsequent stabilization of the CREB: CBP complex stimulates the expression of target genes by acetylating nucleosomal histone H3 at lysine 27 (AcH3K27), followed by recruitment of RNA polymerase II^19,20^. Within the wide range of light-responsive genes, the clock gene *Per1* and the immediate-early gene *cFos* are targets of pS133-CREB dependent gene activation^21–24^.

Within the eukaryote domain, the filamentous fungus *Neurospora crassa* is the most used model system for studying light-inducible pathways, including entrainment and biosynthesis of photoprotective pigments, to name a few^25–28^. The NGF1: White Collar complex (WCC) regulates the light signaling system in this organism. The WCC consists of a heterodimer formed by the product of the *white collar-1* (*wc-1*) and *white collar-2* (*wc-2*) genes^29–31^. The blue light photoreceptor WC-1 interacts in the dark with the histone acetyltransferase NGF1^32,33^ via an LXXLL consensus sequence, a known motif responsible for the interaction between nuclear receptors and their co-activators^34^. After a light pulse, WC-1 changes conformation and directs NGF1 to the chromatin to acetylate histones. At the same time, it stabilizes the transcriptional factor WC-2 on the promoter of target genes, and target gene expression is promoted^35^.

Structural similarities between the WC-1 and PER2 protein suggest that mammalian PER2 may act in a similar way as WC-1 in *N. crassa*. Both proteins are similar in size and contain 3 PAS domains. They are both essential regulators of the circadian clock and can act as co-activators via LXXLL domains^33,36^. PER2 accumulates in the SCN between zeitgeber time (ZT) 14 and 16, which corresponds to the time window of light-responsiveness that leads to induction of genes such as *Per1*^37^. Since WC-1 is involved in light-dependent responses, and due to the resemblance of PER2 with the fungal protein, we wondered whether the mammalian clock factor would modulate the light-mediated CREB signaling pathway to induce genes such as *Per1* or *cFos* in a similar fashion. This resembles the function of WC-2, which is the transcription factor that modulates light responses in *N. crassa*.

Here, we describe a novel function of PER2 as a mediator of the light-dependent CREB signaling in the early night. Using an *in vitro* (forskolin-induced cell lines) and an *in vivo* system (light-stimulated mice), we show that PER2 modulates the light/forskolin mediated assembly of the pSer133-CREB/CRTC1/CBP complex. In particular, we show that PER2 gates the CREB: CRCT1 complex to the early night. Förster resonance energy transfer (FRET) analysis revealed that the complex formation between CREB and CBP depends on the presence of PER2. Consequently, PER2 is important for the histone H3 acetylation at lysine 27 by CBP, and hence induction of *Per1* gene expression. We conclude that PER2 is a scaffold facilitating the formation of the CREB: CBP complex after a light pulse or forskolin stimulus.

## Results

###  PER2 modulates light/forskolin-induced *Per1* and *cFos* gene expression in an opposite manner

**Figure 1:**
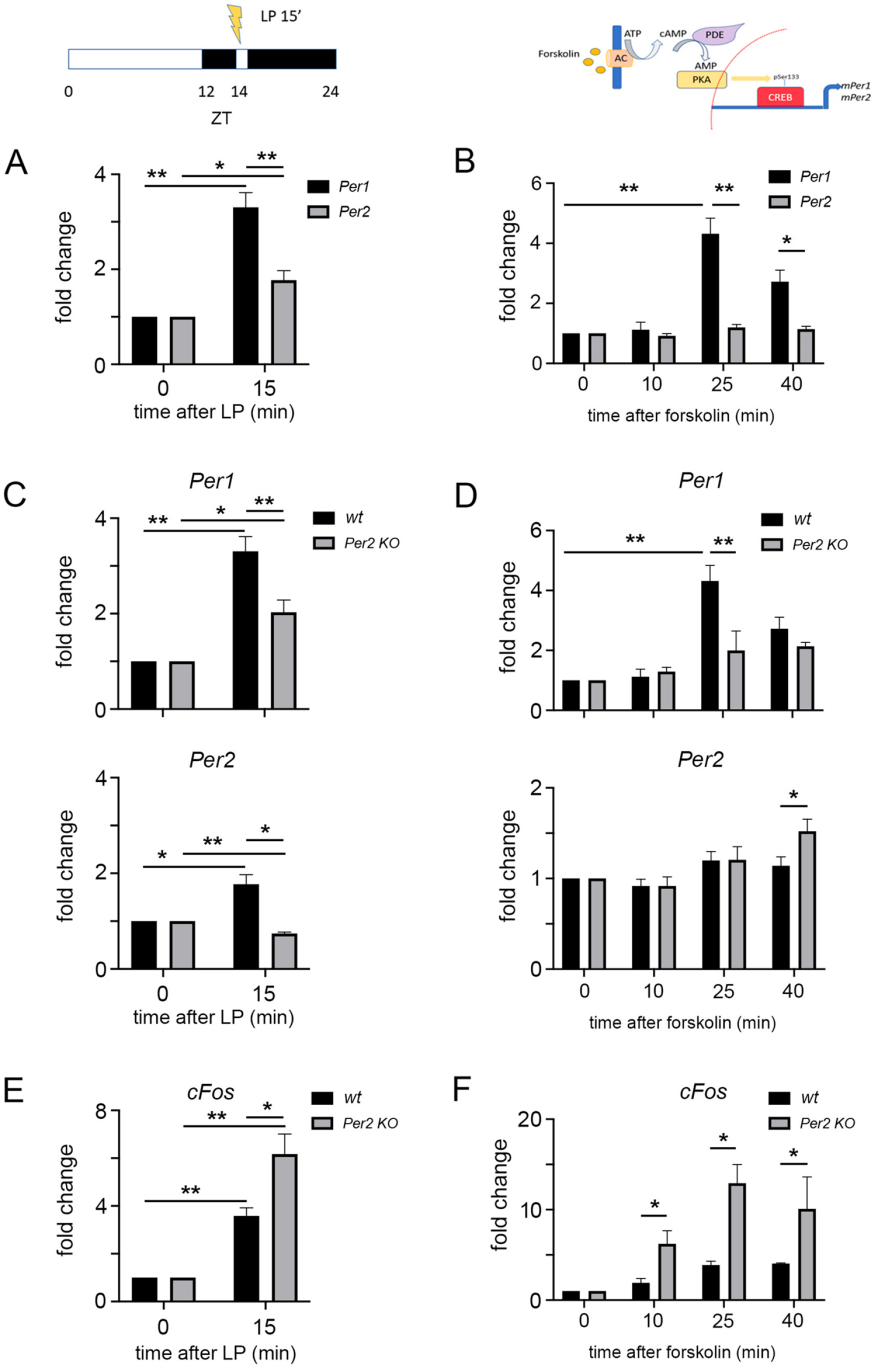
Induction of *Per1*, *Per2*, and *cFos* gene expression after light or forskolin stimulation. The diagram top left indicates the light treatment protocol of mice (**A**, **C**, **E**) and the top right diagram shows the forskolin treatment of cells (**B**, **D**, **F**). (**A**) Fold change of *Per1* (black bars) and *Per2* (grey bars) pre-mRNA expression in the SCN after a 15 min light pulse (LP) applied at ZT14. Values are the mean ± SEM. Student's t-test with Welch's correction, n = 3, *p < 0.05, **p < 0.01. (**B**) Fold change of *Per1* (black bars) and *Per2* (grey bars) pre-mRNA expression in NIH 3T3 cells after forskolin treatment. Values are the mean ± SD. Student's t-test with Welch's correction, n = 3, *p < 0.05, **p < 0.01. (**C**) Fold change of *Per1* (top panel) and *Per2* (bottom panel) pre-mRNA expression in the SCN of wild type (wt, black bars) and *Per2* knock-out (KO, grey bars) mice after a 15 min light pulse (LP) applied at ZT14. Values are the mean ± SEM. Student's t-test with Welch's correction, n = 3, *p < 0.05, **p < 0.01. (**D**) Fold change of *Per1* (top panel) and *Per2* (bottom panel) pre-mRNA expression in immortalized wt or *Per2* KO fibroblast cells after forskolin treatment. Values are the mean ± SD. Student's t-test with Welch's correction, n = 3, *p < 0.05, **p < 0.01. (**E**) Fold change of *cFos* expression in the SCN of wt (black bars) and *Per2* KO (grey bars) mice after a 15 min light pulse (LP) applied at ZT14. Values are the mean ± SEM. Student's t-test with Welch's correction, n = 3, *p < 0.05, **p < 0.01. (**F**) Fold change of *cFos* expression in immortalized wt or *Per2* KO fibroblast cells after forskolin treatment. Values are the mean ± SD. Student's t-test with Welch's correction, n = 3, *p < 0.05.

A light pulse (LP) applied to mammals in the dark phase of a 12 h light: 12 h dark cycle, where zeitgeber time (ZT) 0 is lights on and ZT12 is light off, elicits phase-shifts in locomotor activity^10,38^ paralleled by induction of *Per1* gene expression^6,39^. Here we measured the expression of pre-mRNA of *Per1* and *Per2* genes in the SCN of wild type (wt) mice before and after an LP of 15 min duration at ZT14 (Fig. 1A). The pre-mRNA of *Per1* was strongly induced after the LP, whereas the pre-mRNA of *Per2* was significantly less responsive (Fig. 1A). To corroborate this observation *in vitro*, we stimulated NIH3T3 cells with forskolin, which mimics the effect of phase-shifting via activation of the Protein kinase A (PKA) signaling pathway^40^. We observed that *Per1*, but not *Per2,* pre-mRNA was induced after 25 and 40 min of forskolin treatment (Fig. 1B). These results suggested that the *Per2* gene is less inducible by light or forskolin than *Per1*. Because *Per2* KO mice do not phase delay after a light pulse at ZT14^41^, our general question was whether PER2 was an upstream factor rather than a downstream target of stimulus-mediated clock resetting.

To challenge this hypothesis, we analyzed *Per1* pre-mRNA expression in SCN samples obtained from wt and *Per2* knock-out (KO) mice^42^ collected either in the dark or 15 min after LP at ZT14. We observed that in *Per2* KO mice the light inducibility of *Per1* is dampened compared to wt (Figs. 1C, S1A upper panel). Although *Per2* pre-mRNA was less light-inducible compared to *Per1*, it was not inducible in the *Per2* KO mice (Figs. 1C, S1A lower panel). Subsequently, we analyzed *Per1* and *Per2* pre-mRNA expression in fibroblast cells derived from wt or *Per2* KO animals. After forskolin treatment of the cells we observed induction of *Per1* but not *Per2* after 25 min in wild-type cells (Figs. 1D, S1B, upper panel). In contrast, *Per1* was not induced in *Per2* KO cells, but *Per2* was slightly increased after 40 min (Figs. 1D, S1B, lower panel). These results are in line with the observation that in the SCN *Per1* induction is affected by the loss of *Per2*. Taken together, our observations suggest that PER2 is important for the regulation of the light- and forskolin-dependent induction of *Per1* expression*.* Of note, other clock genes did not respond in the same way as *Per1* after both light and forskolin stimuli, in vivo as well as in vitro (Fig. S1C–D).

Because *cFos* is an immediate-early gene (IEG) and can be induced by light in the SCN^23,24^, we wanted to test whether *cFos* induction may also be affected by the lack of *Per2*. In the SCN, we observed an induction of *c-Fos* after an LP in wt animals and surprisingly an even stronger induction in *Per2* KO mice (Figs. 1E, S1E). Similarly, forskolin treatment caused stronger *cFos* induction after forskolin in *Per2* KO fibroblasts than in wt cells (Figs. 1F, S1F). In order to corroborate our results, we tested the transcriptional activation of other CREB/CBP targets such as *Icer*, *Nor1* and *Nur77*. We observed that *Icer* and *Nor1* transcriptional activation after forskolin treatment was comparable to the activation of *cFos* in wt and *Per2* KO cells (Fig. S1G, H). In contrast, transcriptional activation of *Nur77* was comparable to the activation of *Per1* (Fig. S1I).

These results suggest that PER2 plays an important role in regulating light/forskolin responsive genes, which is different from its role as a circadian regulator. Furthermore, *Per2* can modulate light/forskolin responses in a positive (e.g., *Per1, Nur77*) or negative (e.g., *cFos, Icer, Nor1*) fashion.

### PER2 physically interacts with CREB and modulates its binding to a CRE element in the *Per1* regulatory region

**Figure 2:**
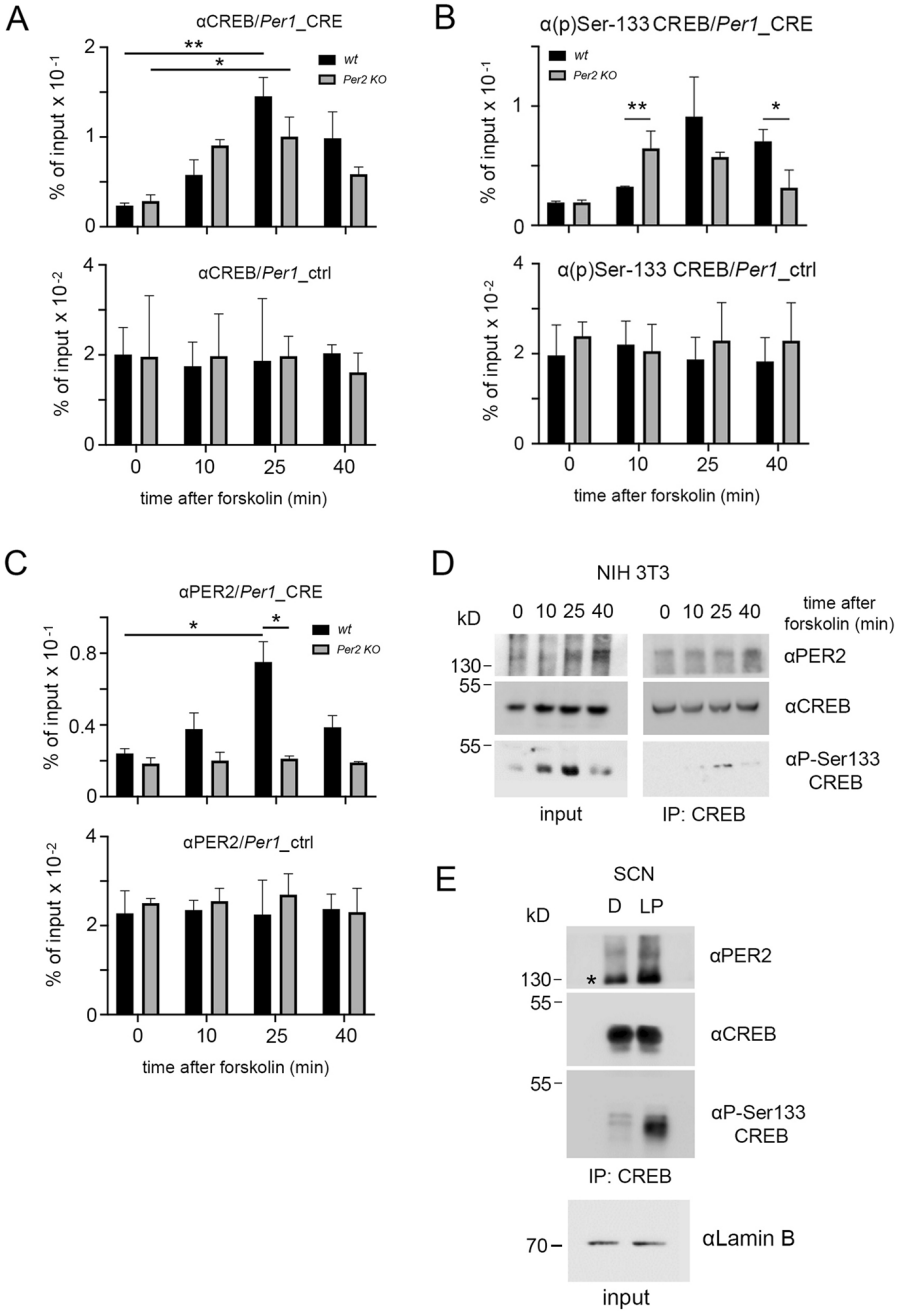
Binding of CREB and PER2 to the CRE-element of the *Per1* promoter and interaction between CREB and PER2. (**A**) Top panel: Chromatin immunoprecipitation (ChIP) of CREB on the CRE-element of the *Per1* promoter in wt (black bars) and *Per2* KO (grey bars) fibroblast cell lines after forskolin treatment. Bottom panel: Control ChIP on an unrelated promoter region of *Per1.* Values are the mean ± SD. Student's t-test with Welch's correction, n = 3, *p < 0.05, **p < 0.01. Two-way ANOVA indicates a significantly different time profile between the two genotypes, **p < 0.01. (**B**) Top panel: ChIP of pSer-133 CREB on the CRE-element of the *Per1* promoter in wt (black bars) and *Per2* KO (grey bars) fibroblast cell lines after forskolin treatment. Bottom panel: Control ChIP on an unrelated promoter region of *Per1.* Values are the mean ± SD. Student's t-test with Welch's correction, n = 3, *p < 0.05. Two-way ANOVA indicates a significantly different time profile between the two genotypes, **p < 0.01. (**C**) Top panel: ChIP of PER2 on the CRE-element of the *Per1* promoter in wt (black bars) and *Per2* KO (grey bars) fibroblast cell lines after forskolin treatment. Bottom panel: Control ChIP on an unrelated promoter region of *Per1.* Values are the mean ± SD. Student's t-test with Welch's correction, n = 3, *p < 0.05. Two-way ANOVA indicates a significantly different time profile between the two genotypes, **p < 0.01. (**D**) Western blot (left panel) was performed on 10% of the total input used for the immunoprecipitation using a CREB antibody as bait (right panel). PER2 co-precipitates with CREB in NIH 3T3 cell extracts, the pSer-133 antibody was used to confirm the forskolin induction. kD = kilo Dalton. (**E**) IP with an antibody recognizing CREB in SCN extracts, which co-precipitated with PER2 before (**D**) and after a 15 min light pulse (LP) at ZT14. The pSer-133 antibody was used to confirm light induction. Input control with lamin B (bottom panel), kD = kilo Dalton. *Unspecific band recognized by the αPER2 antibody in tissue extracts.

Light and forskolin activate signaling pathways that lead to CREB phosphorylation at serine 133 (Ser-133) and subsequently evoke target gene expression such as *cFos* and *Per1*^10–12^. The *Per1* gene contains a CREB response element (CRE)^14^ (Fig. S2A) and is regulated by CREB^14,43^. Since the induction of *Per1* gene expression by light or forskolin is affected by the presence or absence of the *Per2* gene (Fig. 1C, D), we set out to test whether the PER2 protein affects CREB binding to the *Per1* promoter. For this purpose, we studied the CRE-element present in the *Per1* regulatory region, including intron 1 (Fig. S2A) that was shown to be functional^14^. We applied forskolin to wt and *Per2* KO fibroblast cell lines and collected samples at various time points. Then, we performed chromatin immunoprecipitation (ChIP) using an antibody against CREB, followed by RT-qPCR to measure CREB occupancy at the CRE-element of *Per1* and an unrelated element in the *Per1* promoter as control (Fig. S2A). We found that forskolin modulated CREB occupancy at the intronic CRE-element of the *Per1* gene in a time-dependent fashion with a peak at 25 min after the stimulus. On the other hand, this time-dependent change was absent in the control region (Fig. 2A, black columns). This transient CREB occupancy increase mirrored the *Per1* pre-mRNA profile observed in Fig. 1B. Hence, it appeared that CREB was not recruited in a constant fashion to the *Per1* promoter but bound the chromatin upon specific stimulation. Surprisingly, the lack of *Per2* led to an increase of CREB binding to the *Per1* promoter already after 10 min (Fig. 2A, grey columns), but did not further increase after 25 min. This indicated that the two profiles of the occupancy of the regulatory region were different between the two genotypes (two-way ANOVA, *p* < 0.01). Taken together, these results indicate that PER2 modulates CREB recruitment onto the chromatin of *Per1* in a stimulus-dependent manner.

Next, we asked whether PER2 could affect CREB recruitment to the *Per1* gene regulatory region via modulation of CREB phosphorylation at serine 133 (Ser-133), which is associated with light or forskolin-induced phase shifts^44^. Therefore, we performed a ChIP assay using the pSer133-CREB specific antibody as a bait. We observed that this phosphorylated form of CREB was recruited to chromatin in a similar manner as we observed when using the available CREB antibody for both wt (Fig. 2B, black columns) and *Per2* KO (Fig. 2B, grey columns) derived chromatin. This result suggests that lack of *Per2* did not alter the CREB binding profile to chromatin via changes in phosphorylation of Ser-133.

Above, we described that the lack of the PER2 protein affected the recruitment dynamics of CREB to the CRE-element of the *Per1* gene regulatory region. This effect could be of direct or indirect nature. In order to test this, we performed a ChIP assay on the same genomic region using a PER2 specific antibody. Our experiments revealed that the PER2 profile of recruitment to the chromatin (Fig. 2C) was similar to the one for CREB (Fig. 2A) with a maximum at 25 min after forskolin. Interestingly, the circadian clock component BMAL1 was not recruited to the *Per1* CRE element in response to forskolin (Fig. S2B). This observation indicated that the recruitment of PER2 was not due to a general effect on the circadian clock.

Since CREB and PER2 show the same chromatin binding profile upon forskolin stimulation, we wondered whether they might physically interact. We stimulated fibroblast cells with forskolin and collected samples at various time points. Subsequently, we performed a Western blot (WB) and adjusted the protein loading to CREB accumulation. A proper forskolin induction was confirmed by monitoring CREB phosphorylation at serine 133, which was detected with a peak about 25 min after the forskolin stimulus (Fig. 2D left panel). An immunoprecipitation assay (IP) followed by WB revealed that PER2 binds CREB independently from the forskolin induction and that the interaction is specific (Fig. 2D, right panel, Fig. S2C). Next, we investigated whether we could observe this interaction in vivo in SCN tissue of mice. We immunoprecipitated CREB from SCN protein extracts collected in the dark or 15 min after LP, followed by immunodetection. We observed that PER2 specifically binds CREB (Fig. S2D) and that this was independent of the LP and of CREB phosphorylation (Fig. 2E), as evidenced by the PER2 signal in the absence of the pSer133 signal in the dark. Our *in vitro* and *in vivo* data suggest that PER2 physically interacts with CREB and it does so independently from the stimulus (forskolin or light). Therefore, we hypothesize that this interaction influences CREB occupancy in the *Per1* regulatory region at the CRE element.

### PER2 modulates CREB: CRTC1 interaction upon light or forskolin stimulation

CREB occupancy of CRE elements in various promoters has been described to involve a family of factors called CRTCs. It has been demonstrated that in particular CRTC1 localized to the nucleus and dimerized with CREB after exposure to cAMP or calcium^45^. Interestingly, CRTC1 is the main CREB co-activator regulating light responses in the SCN. Upon an LP, CRTC1 was observed to translocate into the nucleus to support CREB activity^46^. These observations prompted us to investigate whether the light or forskolin mediated effect on the induction of the *Per1* gene involved CREB: CRTC1 dimerization and whether PER2 modulated this complex.

**Figure 3:**
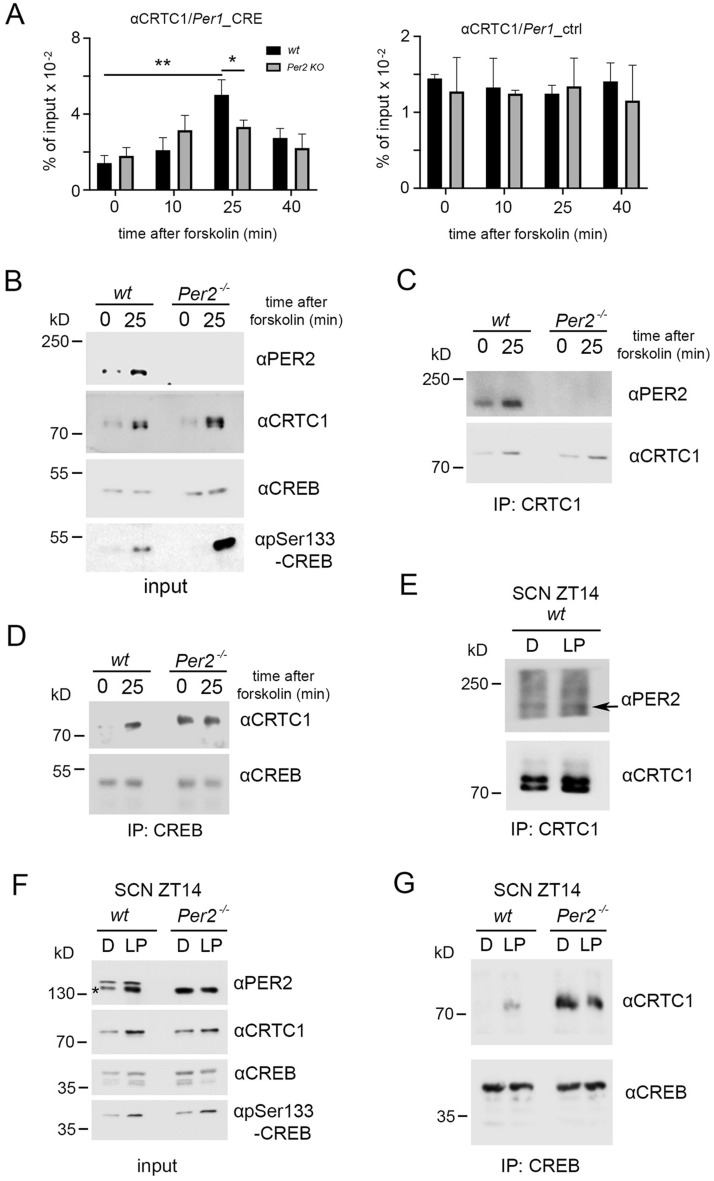
Binding of CRTC1 to the *Per1* promoter and interaction with PER2 and CREB. (**A**) Left panel: Chromatin immunoprecipitation (ChIP) of CRTC1 on the CRE-element of the *Per1* promoter in wt (black bars) and *Per2* KO (grey bars) fibroblast cell lines after forskolin treatment. Right panel: Control ChIP on an unrelated promoter region of *Per1.* Values are the mean ± SD. Student's t-test with Welch's correction, n = 3, *p < 0.05, **p < 0.01. Two-way ANOVA indicates a significantly different time profile between the two genotypes, **p < 0.01. (**B**) Western blot of wt and *Per2*^-/-^ (*Per2* KO) cells before and 25 min after forskolin treatment. CRTC1 is induced by forskolin independently of *Per2*. CREB is phosphorylated (pSer133) in both genotypes after the stimulus. (**C**) Immunoprecipitation (IP) of CRTC1 pulls down PER2 independently of forskolin treatment. (**D**) IP of CREB pulls down CRTC1 after forskolin treatment in wt cells, but in *Per2*^-/-^ cells, the interaction between CREB and CRTC1 is forskolin independent. (**E**) IP of CRTC1 in SCN extracts before and after a 15 min light pulse (LP) at ZT14. Co-IP of PER2 is independent of LP. (**F**) Western blot of wt and *Per2*^-/-^ SCN tissue performed as in B on samples collected before and after LP, * unspecific band recognized by the αPER2 antibody in tissue extracts. CREB is phosphorylated in both genotypes after the light pulse. (**G**) IP of CREB pulls down CRTC1 in wt SCN extracts after LP, but in *Per2*^-/-^ SCN the CREB: CRTC1 interaction is light-independent. kD = kilo Dalton.

We applied forskolin to wt and *Per2* KO fibroblast cell lines and collected samples at various time points. Then, we performed chromatin immunoprecipitation (ChIP) using an antibody against CRTC1, followed by RT-qPCR to measure CRTC1 occupancy at the CRE-element of *Per1* and an unrelated element in the *Per1* promoter as control (Fig. 3A). We observed that in wt cells, CRTC1 recruitment to the *Per1* intronic chromatin after forskolin treatment displayed a profile comparable to CREB (Fig. 2A, black columns) with a peak at 25 min after forskolin treatment (Fig. 3A, black columns). Conversely, in *Per2* KO cells, the recruitment of CRTC1 to the *Per1* promoter after forskolin application was reduced (Fig. 3A, grey column) and comparable to the CREB profile (Fig. 2A, grey columns). Thus, PER2 appeared to influence CREB and CRTC1 recruitment to the *Per1* CRE element in a similar way.

Next, we investigated whether CRTC1 abundance was affected by the lack of PER2. The WB with samples obtained from wt and *Per2* KO cell lines collected before and 25 min after forskolin stimulation did not show any difference in CRTC1 levels between the two genotypes. The induction mediated by forskolin increased CRTC1 amounts in both genotypes in a similar manner (Fig. 3B). Interestingly, the interaction between CREB and CRTC1 appeared to be increased by the forskolin stimulus (about 20% of the total input), as shown by immunoprecipitation (IP) using an anti-CREB antibody as bait (Fig. S3A). In the absence of a good knock-out cell line as negative control, we validated the result repeating the IP using either CREB or rabbit IgG (negative control) or TORC-1(CRTC-1, positive reverse CoIP) antibodies as bait. We observed that the interaction between CREB and CRTC-1 is specific, because there was no signal in the IP when the rabbit IgG antibodies were used (Fig. S3B). We wondered whether PER2 might affect CREB: CRTC1 interaction. IP with an anti-CRTC1 antibody as bait revealed that PER2 interacted with CRTC1 independently from the forskolin stimulus (Fig. 3C). Hence, PER2 binds to both CREB (Fig. 2D, E) and CRTC1 (Fig. 3C), although CREB and CRTC1 interact only after a stimulus^45^ (Fig. S3A). To test whether PER2 could modulate the CREB: CRTC1 interaction, we immunoprecipitated CREB in both wt and *Per2* KO cell lines, without and 25 min after forskolin treatment. Surprisingly, CREB and CRTC1 interacted after this treatment only in wt cells. In contrast, in the *Per2* KO cells (*Per2*^-/-^), the interaction was stimulus-independent (Fig. 3D), as evidenced by the co-IP of both components before forskolin treatment.

Next, we investigated whether our observations in cells were also manifested in the SCN of mice. Similar to what we observed in the fibroblast cell line, CREB interacted with CRTC1 only after the light stimulus in SCN samples (Fig. S3C). Immunoprecipitation experiments showed that PER2 interacted with CRTC1 in a specific manner (Fig. S3D). This interaction was independent of an LP, as revealed when sample loading between the two conditions was adjusted to equal amounts of CRTC1 (Fig. 3E). Furthermore, light-induced CRTC1 accumulation was not affected by the lack of PER2 (Fig. 3F), and CREB was still phosphorylated at Ser-133 after the LP, as revealed by WB (Fig. 3F). Although CREB phosphorylation and CRTC1 accumulation were unaffected by PER2, the CREB: CRTC1 interaction was modulated by the clock factor (Fig. 3G) in a comparable manner as observed in cells (Fig. 3D) when a stimulus (light or forskolin) was applied. Additionally, CREB and CRTC1 interacted before the LP in *Per2* KO mice (Fig. 3G) which was also observed in cells before the forskolin stimulus (Fig. 3D). Altogether these results define PER2 as a modulator of CREB: CRTC1 interaction. This modulation depends on specific triggers, such as forskolin for cell cultures or light for the SCN cells in animals.

### PER2 mediates CREB: CBP interaction upon light or forskolin stimulation

**Figure 4:**
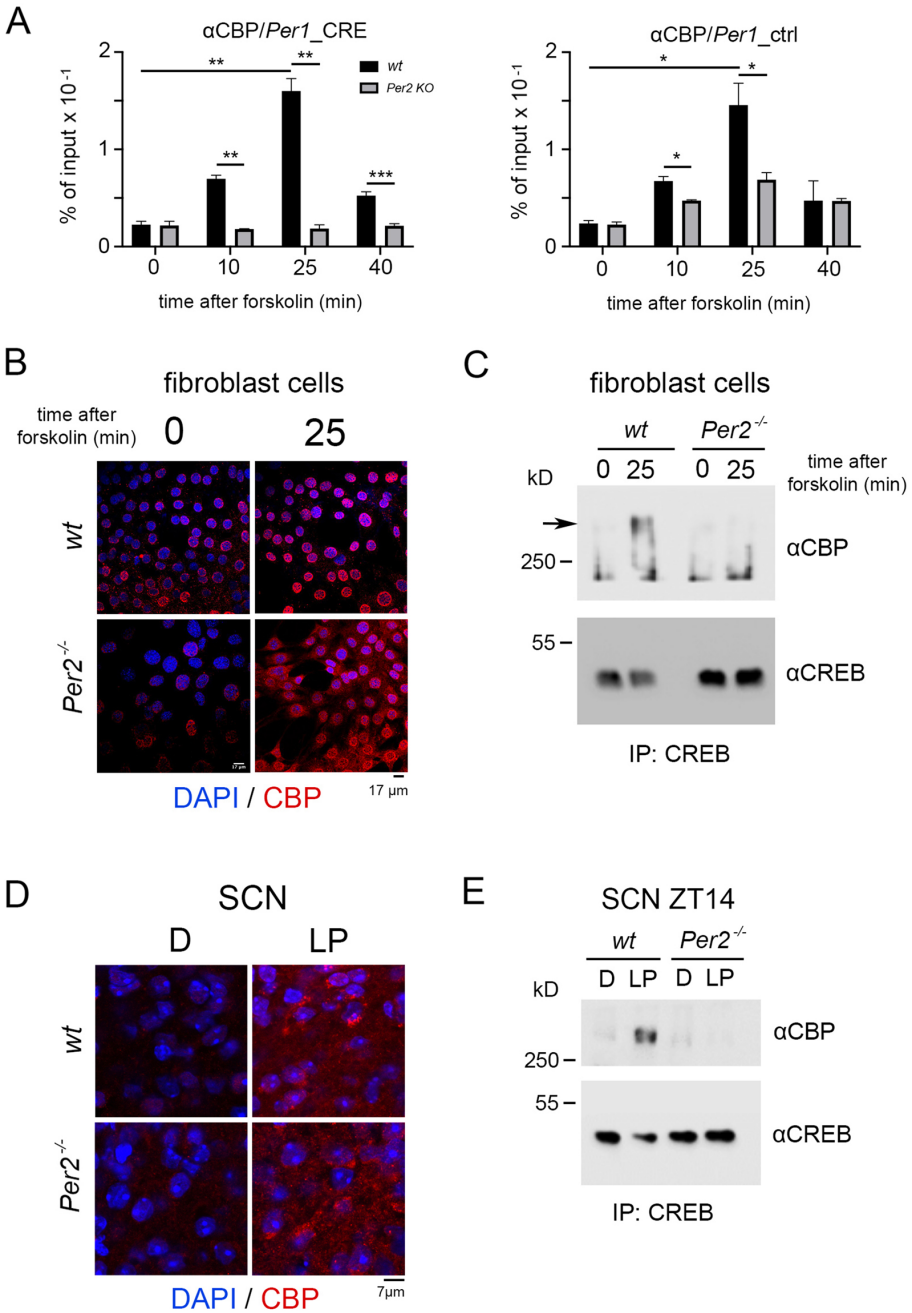
Modulation of the interaction between CREB and CBP by PER2. (**A**) Left panel: Chromatin immunoprecipitation (ChIP) of CBP on the CRE-element of the *Per1* promoter in wt (black bars) and *Per2* KO (grey bars) fibroblast cell lines after forskolin treatment. Right panel: Control ChIP on a promoter region of *Per1* without a CRE-element*.* Values are the mean ± SD. Student's t-test with Welch's correction, n = 3, *p < 0.05, **p < 0.01. Two-way ANOVA indicates a significantly different time profile between the two genotypes, left panel: ****p < 0.0001, right panel: **p < 0.01. (**B**) Immunohistochemistry (IHC) of wt and *Per2*^-/-^ cells before and after forskolin treatment. CBP (red signal) is induced after forskolin treatment in both genotypes. Scale bar: 17 µm. (**C**) IP of CREB pulls down CBP in wt but not *Per2*^-/-^ cells after forskolin treatment, kD = kilo Dalton. (**D**) IHC of wt and *Per2*^-/-^ SCN before and after a light pulse (LP) at ZT14. CBP (red signal) is induced after LP in both genotypes. Scale bar: 7 µm. (**E**) IP of CREB pulls down CBP in wt SCN extracts after LP, but not in *Per2*^-/-^ SCN extracts. kD = kilo Dalton.

CREB binding protein (CBP) is a histone acetyltransferase that can heterodimerize with CREB when CREB is phosphorylated at Ser-133. This CREB: CBP complex then promotes gene expression^18^. To determine the CBP recruitment profile to the CRE-element of the *Per1* regulatory region we performed a ChIP assay using an antibody against CBP, followed by RT-qPCR (Fig. 4A). In wt animals, recruitment of CBP to the CRE-element was very similar (Fig. 4A, left panel, black columns) to the profiles observed for CREB (Fig. 2A), pSer-133 CREB (Fig. 2B), PER2 (Fig. 2C), and CRTC1 (Fig. 3A). Interestingly, CBP appeared to bind to the control region in the *Per1* promoter as well (Fig. 4A, right panel, black columns) possibly due to interaction with BMAL1^47^, since this region contained a BMAL1 binding site (Fig. S2B). In contrast, in *Per2* KO cells, CBP did not bind to the CRE-element of *Per1* and to a reduced extent to the control element after forskolin treatment compared to background signal at time point 0 (Fig. 4A, grey columns). These results suggested that PER2 affected CBP binding to both *Per1* promoter regions.

Since we were interested in the CREB mediated regulation of the *Per1* gene, we wondered how PER2 could affect CBP recruitment to the CRE element. We investigated whether PER2 could favor accumulation of CBP in the nucleus. We tested the cells for CBP expression before and after forskolin treatment (Fig. 4B). We saw that CBP accumulation in the nuclei was increased after forskolin treatment, but no distinct difference between the two genotypes was observed (Fig. 4B). We performed immunofluorescence on wt and *Per2* KO fibroblasts to evaluate the specificity of our PER2 antibody (Fig. S4A). Next, we tested whether forskolin treatment could induce Ser133 phosphorylation of CREB in our assay using an anti-pSer133 CREB antibody. The phosphorylation of CREB was induced as expected in both wt and *Per2* KO cells 25 min after but not before forskolin treatment (Fig. S4B). Since CBP can still accumulate in the nuclei after forskolin induction in the absence of *Per2*, we investigated whether PER2 could affect the interaction between CBP and CREB. This interaction is essential for the CBP acetyltransferase activity on chromatin. Therefore, we immunoprecipitated CREB at 0 and 25 min after forskolin treatment of cells and performed IP using an antibody against CREB followed by WB (Fig. 4C). We observed that CBP was co-precipitated with CREB 25 min after forskolin treatment.

In contrast, this co-precipitation was not observed in *Per2* KO cells (Fig. 4C). Subsequently, we wanted to see whether a *Per2*-dependent CREB: CBP interaction could also be observed in the SCN of mice after the LP stimulus. Immunohistochemistry on SCN tissue showed that the amount of CBP was increased after LP in both wt and *Per2* KO tissue, indicating that lack of *Per2* did not affect the increase of CBP levels in response to LP (Fig. 4D, control Fig. S4C). Next, we immunoprecipitated CREB from SCN extracts collected in the dark and after LP at ZT14 of both genotypes (Fig. 4E). Similar to the observation in cells (Fig. 4C), CBP was co-precipitated with CREB in wt extracts but not in extracts of *Per2* KO mice. Taken together, our findings indicate that PER2 is necessary for the formation of the CREB: CBP complex after a stimulus such as forskolin or a light pulse.

### FRET analysis indicates that PER2 supports the interaction of CREB and CBP domains

**Figure 5:**
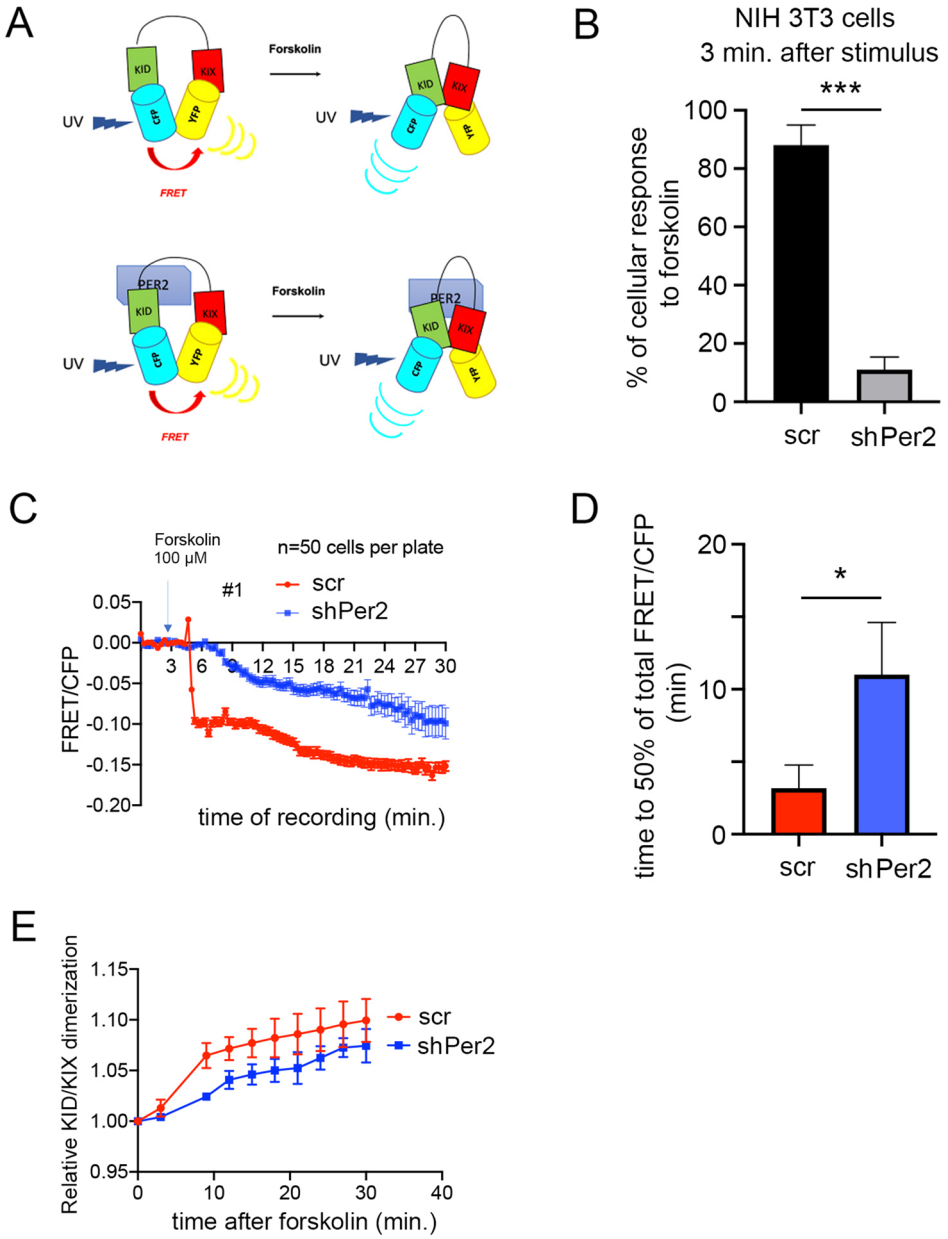
FRET analysis of the interaction between PER2 and the KID/KIX domains of CREB/CBP. (**A**) Top panel: Schematic diagram of the ICAP construct that is an indicator of CREB: CBP dimerization due to phosphorylation on the KID (CREB) domain at serine 133. Bottom panel: Proposed model of PER2 mediated interaction between the KID and KIX domains. Modified from^48^ using PowerPoint software for Mac version 16.16.27 (**B**) % of cellular response to forskolin after 3 min in NIH 3T3 cells transfected either with scrambled (scr) shRNA or an shRNA directed against *Per2* (shPer2). Values are the mean ± SD. Student's t-test with Welch's correction, n = 3, ***p < 0.001. (**C**) The FRET/CFP signal ratio changes in response to forskolin treatment in cells transfected with either a scr control (red) or shPer2 (blue) expressing construct. Values are the mean ± SD. Two-way ANOVA revealed a significant difference between the curves, n = 50, ****p < 0.0001. (**D**) Time elapsed to reach half of the total FRET/CFP signal is significantly shorter in scr control transfected cells than shPer2 transfected cells. Values are the mean ± SD. Student's t-test with Welch's correction, n = 50, *p < 0.05. (**E**) KID/KIX dimerization is significantly lower in shPer2 cells than scr control cells. Values are the mean ± SD. Two-way ANOVA, n = 50, *p < 0.05.

The results presented above lend support to the notion that PER2 serves as a scaffold for CREB: CBP interaction. To further challenge this idea, we performed Förster resonance energy transfer (FRET) experiments, a widely used method to investigate molecular interactions between proteins such as CREB: CBP in living cells^48^. We used a sensor called ICAP (an indicator of CREB activation due to phosphorylation). The sensor is composed of three different elements: 1) the KID domain of CREB containing the Ser-133, which is phosphorylated upon forskolin induction, 2) the KIX domain of CBP, which is responsible for the dimerization with phospho-CREB and 3) a short linker that separates the KID from the KIX domain. KID is flanked by a cyan fluorescent protein (CFP), while KIX is flanked by a yellow fluorescent protein (YFP). When KID is not phosphorylated, the ICAP conformation allows CFP to transfer energy to YFP, producing FRET resulting in yellow light emission. After a stimulus (forskolin), the serine in KID is phosphorylated and binds to KIX. The dimerization separates CFP from YFP, leading to decreased FRET resulting in blue light emission (Fig. 5A upper panel).

We hypothesized that depletion of the endogenous PER2 could affect the KID: KIX dimerization (Fig. 5A bottom panel). To test this hypothesis, we co-transfected ICAP and either scrambled (scr), or *Per2* directed shRNA (shPer2) into NIH3T3 cells. Subsequently, we acquired the FRET signal. 3 min after forskolin treatment, which is the standard latency time for FRET signal detection, we noted that only about 10% of shPer2 transfected cells (*Per2* knock-down (KD)) were responsive to the stimulus compared to about 90% of scr transfected cells (Fig. 5B). As evidenced by single-cell traces (Fig. S5A), most shPer2 transfected cells were not responsive, but some with a substantial delay, compared to the scr controls. These results suggested that the knock-down of *Per2* affected the KID: KIX interaction. Quantification of the FRET signal acquired over 30 min from 50 cells showed a difference in the profiles of scr and shPer2 transfected cells (Figs. 5C, S5B). 50% of the scr control cells responded to the forskolin treatment within about 3 min, while shPer2 cells needed over 10 min (Fig. 5D). Additionally, the relative KID: KIX dimerization was significantly higher in scr control cells compared to shPer2 cells up to around 20 min after the stimulus (Fig. 5E). Altogether these results reinforce our notion that PER2 may support the CREB: CBP dimerization via their respective KID: KIX domains.

### PER2 modulates CBP-mediated chromatin acetylation at lysine 27 of histone H3 after forskolin or light stimulation

**Figure 6:**
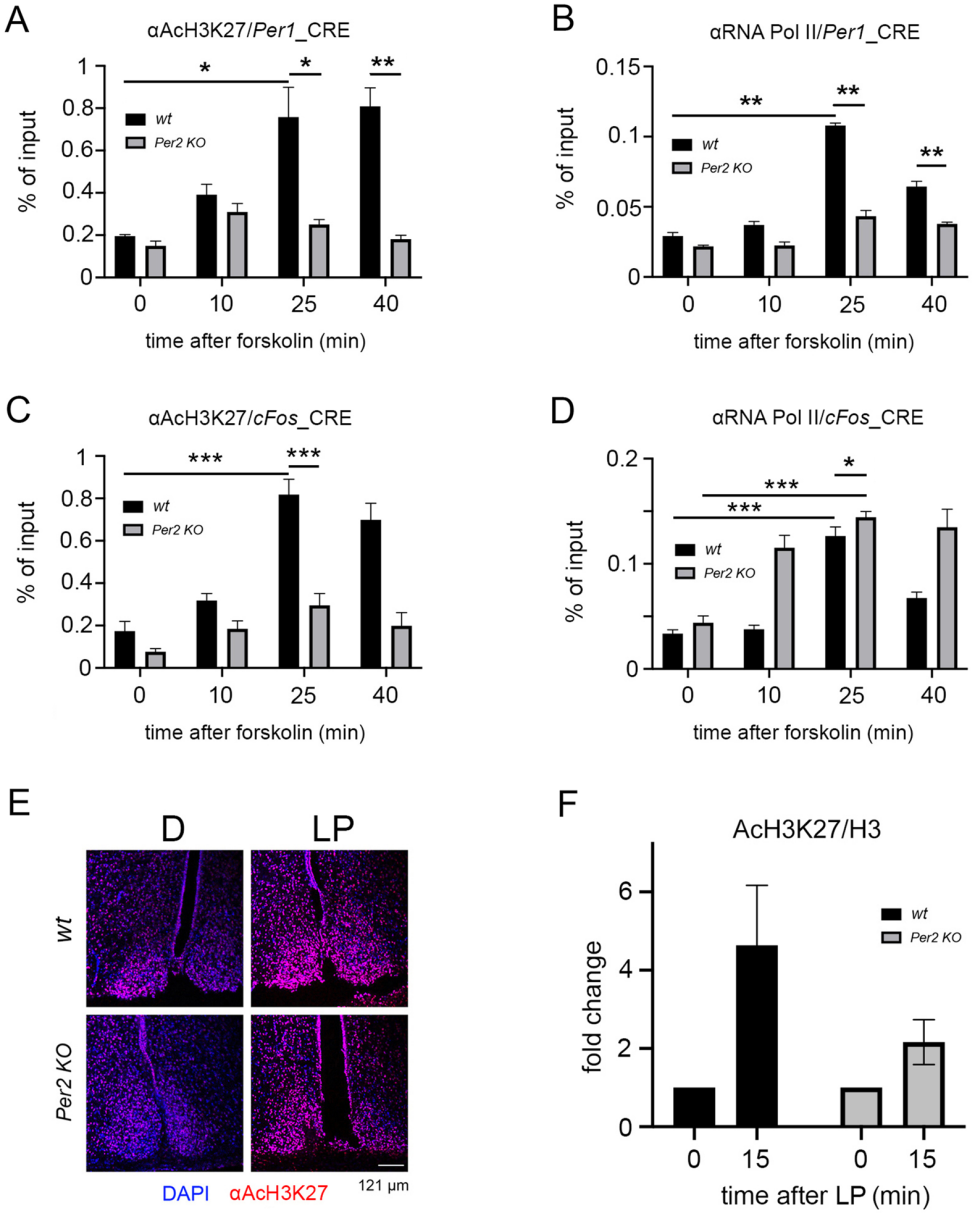
Modulation of CBP-mediated chromatin acetylation of histone H3K27 by PER2. (**A**) Chromatin immunoprecipitation (ChIP) of acetylated histone H3 (AcH3K27) on the CRE-element of the *Per1* promoter in wt (black bars) and *Per2* KO (grey bars) fibroblast cell lines after forskolin treatment. Values are the mean ± SD. Student's t-test with Welch's correction, n = 3, *p < 0.05, **p < 0.01. Two-way ANOVA indicates a significantly different time profile between the two genotypes, **p < 0.01. (**B**) The ChIP of RNA polymerase II (RNA Pol II) on the CRE-element of the *Per1* promoter in wt (black bars) and *Per2* KO (grey bars) fibroblast cell lines after forskolin treatment. Values are the mean ± SD. Student's t-test with Welch's correction, n = 3, **p < 0.01. Two-way ANOVA indicates a significantly different time profile between the two genotypes, ***p < 0.001. (**C**) ChIP with an antibody against AcH3K27 on the Cre-element of the *cFos* promoter in wt (black bars) and *Per2* KO (grey bars) fibroblast cell lines after forskolin treament. Values are the mean ± SD. Student's t-test with Welch's correction, n = 3, ***p < 0.001. (**D**) ChIP of RNA Pol II on the CRE-element of the *cFos* promoter in wt (black bars) and *Per2* KO (grey bars) fibroblast cell lines after forskolin treatment. Values are the mean ± SD. Student's t-test with Welch's correction, n = 3, *p < 0.05, ***p < 0.001. (**E**) Immunohistochemistry (IHC) on wt and *Per2* KO SCN before and after a light pulse (LP) at ZT14. AcH3K27 (red signal) is stronger after LP in wt compared to *Per2* KO SCN. Scale bar: 121 µm. (**F**) Quantification of 3 independent Western blot experiments of wt and *Per2* KO SCN before and after an LP at ZT14. Acetylation of H3K27 is increased after an LP with a tendentially lower increase in *Per2* KO. Two-way ANOVA, *p* = 0.059.

CBP binds to chromatin by dimerizing with pSer133-CREB to exert its histone acetyltransferase (HAT) activity^49^. Lysine 27 (K27) of histone H3 is the main target of CBP HAT activity to acetylate (Ac) K27 of histone H3^50^. We wondered whether this epigenetic modification (AcH3K27) could be observed on chromatin containing the *Per1* CRE-element in cells after forskolin treatment. A ChIP assay, using an anti-AcH3K27 antibody for IP, followed by RT-qPCR revealed that chromatin remodeling at that specific amino acid of histone H3 is forskolin-dependent (Fig. 6A, black columns). We observed highest acetylation of histone H3 in the *Per1* CRE-element containing chromatin between 25 and 40 min after stimulus application (Fig. 6A, black columns). Interestingly, acetylation was dramatically reduced in the *Per2* KO cells (Fig. 6A, grey columns). This observation suggested that CBP needed PER2 as a co-factor for assembling a functional complex allowing acetylation of histone H3 on the *Per1* CRE-element. This observation is the first evidence indicating that acetylation at K27H3 is a result of PER2-dependent forskolin stimulation.

Acetylation of histone H3 leads to the assembly of the RNA polymerase II (RNA Pol II) complex to promote gene transcription^20^. Therefore, we tested whether the formation of such an RNA Pol II containing complex on the *Per1* CRE-element was promoted by forskolin treatment of cells and whether PER2 may be involved in this process. We performed a ChIP assay using an antibody against RNA Pol II as bait. We noted that RNA Pol II recruitment to chromatin of wt cells (Fig. 6B, black columns) paralleled the acetylation dynamics of histone H3 on K27 (Fig. 6A, black columns). In contrast, the recruitment of RNA Pol II was lower in cells lacking *Per2* (Fig. 6B, grey columns), mirroring the absence of acetylation of H3K27 (Fig. 6A, grey columns). These results provide additional evidence for the importance of PER2 as a facilitating component for assembling the stimulus-dependent transcriptional complex on the intronic CRE-element of the *Per1* gene.

PER2 can promote the forskolin-induced gene expression of *Per1*, whereas it can repress that of *cFos* (Fig. 1). Therefore, we wondered how the chromatin of the CRE element in the *cFos* promoter was affected after forskolin treatment of cells. Therefore, we performed a ChIP assay using an anti-AcH3K27 antibody for IP, followed by RT-qPCR. Our results showed that the chromatin acetylation profile mirrored the one obtained on the *Per1* CRE region (Fig. 6A, C). While we observed in wt samples the highest acetylation of histone H3 in the CRE-element containing chromatin between 25 and 40 min after stimulus application (Fig. 6C, black columns), the same acetylation was profoundly dampened in the *Per2* KO samples (Fig. 6C, grey columns). As discussed before histone acetylation leads to the recruitment of RNA Pol II onto the chromatin. Therefore, we tested by ChIP whether forskolin treatment of cells affected the formation of the transcriptional complex including RNA Pol II on the promoter of *cFos* and whether PER2 could affect this formation. We found that RNA Pol II could be recruited onto the *cFos* chromatin of wt cells with a profile mirroring the gene expression (Fig. 6D, black columns). Surprisingly, the recruitment of RNA Pol II onto the *cFos* chromatin in *Per2* KO samples was higher compared to wt (Fig. 6D, grey columns). This is consistent with the observation that the *cFos* pre-mRNA was more abundant in *Per2* KO cells compared to wt (Fig. 1F). Together, our results indicate that the forskolin-mediated *cFos* gene expression is independent of the chromatin acetylation at lysine 27, although no difference was observed between wt and *Per2* KO cells in CREB recruitment to the *cFos* chromatin (Fig. S6A).

A previous study that described light-dependent histone phosphorylation in the SCN of mice^51^ prompted us to ask whether acetylation of histone H3 at K27 in the SCN was light-dependent. We applied an LP at ZT14 to mice as described before and subsequently collected SCN tissue. We performed an immunofluorescence assay using an anti AcH3K27 antibody. At ZT14 H3 in the SCN of wt mice displayed a basal level of chromatin acetylation, which was strongly increased after application of an LP (Fig. 6E upper row). In contrast, the immunofluorescence signal was partially dampened in the SCN of *Per2* KO mice (Fig. 6E lower row). To confirm this observation, we performed WB on pooled SCN tissue collected in the dark or 15 min after LP from both genotypes (Fig. S6B). Quantification confirmed that AcH3K27 was increased right after the light stimulus in wt SCN tissue, whereas the induction was blunted in SCN extracts obtained from *Per2* KO mice (Fig. 6F). Together, these data suggested that the light signaling cascade did regulate chromatin acetylation and that PER2 modulated this process in the SCN.

## Discussion

In the present study, we report a role of the clock protein PER2 as a modulator of the light-dependent CREB signaling in the early night. In particular, we observed that PER2 acted as a positive factor in stimulus dependent *Per1* and *Nur77* gene expression, while it functioned as a negative regulator in *cFos, Icer* and *Nor1* gene induction (Fig. 1C–I). This functional dichotomy indicated that the transcriptional complex involving PER2 is not identical on the *Per1* and *cFos* promoters. That PER2 can act as a positive or negative regulator is consistent with previous observations that this protein has a modulatory influence on transcriptional regulation in both directions in mammals^52,53^. Because deletion of *cFos* in mice attenuated behavioral responses to light only marginally^54^, we focused on the role of PER2 in the stimulus-dependent activation of the *Per1* promoter. Since light activates several signaling pathways in mammals culminating in the induction of the *Per1* gene^6,39^, we investigated the role of PER2 as co-factor in CREB mediated transcription, a common downstream target of protein kinase cascades^18^.

ChIP analysis on the intronic CRE-element of the *Per1* gene (Fig. S2A) revealed that CREB and its known co-factor CRTC1 are recruited in a time-dependent manner after a forskolin stimulus with a peak around 25 min. However, although CREB and CRTC1 bound to the same region in the *Per2* KO cell lines, their binding was less pronounced and occurred earlier, with a peak around 10 min (Figs. 2A, 3A). On the other hand, the histone acetyltransferase CBP and the RNA Pol II were recruited to the same element only in wt, but not in *Per2* KO cells (4A, 6B). Our observations are in agreement with previous findings that describe CREB as an activator of *Per* transcription^14,55^. Interestingly, PER2 could be recruited to the CRE-element of the *Per1* promoter as well, with the same temporal profile as CREB (Fig. 2A, C). These results suggested that PER2 could be part of the CREB-containing transcriptional complex. The hypothesis was further corroborated by the observation of a discrete reduction and temporal profile of CREB and CRTC1 binding to the *Per1* promoter in *Per2* KO cells.

**Figure 7:**
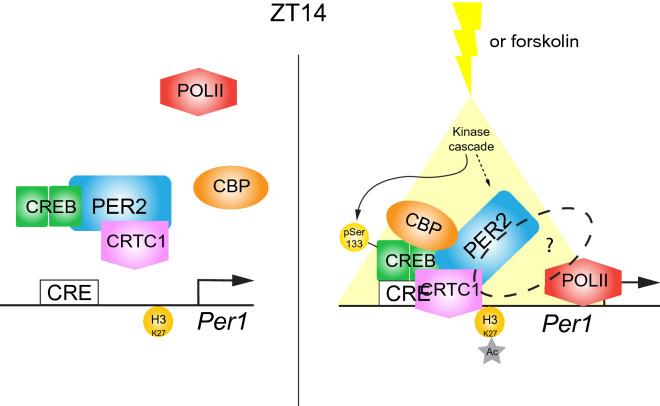
Model of PER2 modulating the assembly of the CREB/CRTC1/CBP transcriptional complex. Left: In the early dark phase at ZT14, PER2 interacts with CREB and CRTC1. On the other hand, CREB and CRTC1 do not dimerize at this stage, and all the elements do not bind to the CRE element in the *Per1* promoter. CBP is not recruited to the regulatory region of *Per1* and the histone H3K27 is not acetylated. *Per1* is not transcribed through this pathway. Right: In the SCN or in cells a light pulse or forskolin treatment initiates a kinase cascade that leads to CREB phosphorylation and assembly of the CREB: CRTC1: CBP complex which is facilitated by PER2. As a consequence of this assembly, histone H3K27 is acetylated (grey star) and the *Per1* gene is transcribed. The hatched oval with question mark indicates that most likely additional factors contribute to the initiation of transcription by the RNA polymerase II (POLII). Figure was generated using Adobe Illustrator version 24.3.1.

Immunoprecipitation experiments revealed that PER2 co-precipitated with CREB (Fig. 2D, E) and with CRTC1 (Fig. 3C, E) in a stimulus-independent manner, suggesting that PER2 could interact with both CREB and CRTC1 before the stimulus occurred (Fig. 7, left). Interestingly, CREB bound to CRTC1 independent of a stimulus when PER2 was absent, however in the presence of PER2 this interaction became stimulus-dependent (Fig. 3D, G). In contrast, interaction between CREB and CBP did not occur in absence of PER2, but appeared in presence of it in a stimulus-dependent fashion (Fig. 4C, E). Hence, a stimulus led to rearrangement of the components CREB, CRTC1 and CBP with PER2 most likely acting as a scaffold to facilitate this rearrangement (Fig. 7, right). Thus, we conclude that PER2 is the factor that mediates the stimulus-dependent build-up of the complex facilitating CRE element-dependent transcription of genes of the *Per1* type family (*Per1*, *Nur77*). The limitation of our observation is, that we do not know whether this is a general mechanism or whether this mechanism is restricted to a subset of CRE elements that are flanked by specific uncharacterized regulatory sequences that are present in the *Per1* and *Nur77* genes. However, the conclusion that PER2 appears to be the stimulus-dependent factor that facilitates gene activation in a subset of CREB dependent target genes seems reasonable. A number of previous observations relate *Per* gene activation to stimulus-dependent behavioral responses such as entrainment by light^41^, response to drugs^56–59^, and adaptation to temperature and humidity^60^. The role of PER2 in the CREB complex appears not to be essential (Fig. 1C). However, PER2 increases the transcriptional activation potential of the CREB complex. Since PER2 protein is expressed in a circadian fashion the transcriptional potential of the CREB complex is guided to a particular time window and boosted. Hence, PER2 seems to be important to increase environmental signal transduction to particular times of the day.

Dynamic chromatin remodeling in the SCN has been suggested to occur in response to a physiological stimulus such as light^51^. These findings are in line with our observation that stimulus-dependent activation of *Per1* transcription involved the acetylation of histone H3 at lysine 27 and that this acetylation depended on the presence of PER2 (Fig. 6A). The acetylation profile on the *Per1* promoter was also mirrored by the recruitment of RNA Pol II and suggested a functional importance of this *Per2* dependent H3K27 modification (Fig. 6B). In contrast, recruitment of RNA Pol II on the *cFos* promoter was independent of *Per2* and the H3K27 modification (Fig. 6C, D). This suggested that another histone modification is important in the CREB mediated transcriptional activation of promoters of the *cFos* type family (e.g. *Icer*, *Nor1*).

The same mechanism as observed in cell cultures on the *Per1* promoter is likely to be present in the SCN (Fig. 6E, F). Of note is the strong decrease of the magnitude of H3K27 acetylation in *Per2* KO SCN (Fig. 6F). This can't be accounted for by an event happening only at the *Per1* genomic locus. It is likely, that PER2 has a more widespread function to promote CBP action in order to acetylate additional genomic loci. Interestingly, light has been described to affect other epigenetic changes in the genome. For example, changes in day length affected the methylation pattern on chromatin in the SCN in a reversible manner^61^. Taken together, it appears that stimuli such as light can affect gene expression via acetylation, methylation, or phosphorylation to modulate transcriptional responses in order to adapt the clock to the current environmental parameters (Fig. 7).

The molecular mechanism that regulates light-dependent responses described here appears to be different from *Drosophila*, but similar to the one described in *Neurospora crassa*. In this organism, the transcriptional complex transducing the light signal is composed of WHITE COLLAR 1 (WC-1), WHITE COLLAR-2, and *Neurospora* GCN5 like-1 (NGF-1)^32,33^. After a light pulse, the large White Collar Complex (WCC) can be rearranged in a similar way as shown in the model we propose here for the mouse (Fig. 7).

Overall our study provides evidence that the PER2 protein can act as a scaffold in the CREB complex. In this role it can influence CREB mediated transcriptional initiation in a positive or negative manner. This most likely depends on additional unknown co-factors necessary for at least two distinct promoter families, one defined by the *Per1* promoter and the other by the *cFos* promoter. Because environmental signals converge in many cases on CREB mediated transcription our model is consistent with the role of PER2 as environmental sensor^41,56,60^. Our observations may be of general nature and will have to be tested on a genome wide scale.

## Materials and methods

### Animals and housing

Mice were housed with food and water ad libitum in transparent plastic cages (267 mm long × 207 mm wide × 140 mm high; Techniplast Makrolon type 2 1264C001) stainless-steel wire lid (Techniplast 1264C116), kept in light- and soundproof ventilated chambers. All mice were entrained to a 12:12 h light:dark (LD) cycle, and the time of day was expressed as Zeitgeber time (ZT; ZT0 lights on, ZT12 lights off). Two- to four-month-old males were used for the experiments. Housing and experimental procedures were performed following the guidelines of the Schweizer Tierschutzgesetz and the declaration of Helsinki. The state veterinarian of the Canton of Fribourg and the national approved ethics committee approved the protocol. The study was carried out in compliance with the ARRIVE guidelines. The floxed *Per2* mice^42^ are available at the European Mouse Mutant Archive (EMMA) strain ID EM: 10599, B6;129P2-Per2tm1Ual/Biat.

### Light pulse and tissue isolation

Light pulse (LP., circa 500 lx) was given at ZT14, and mice were subsequently sacrificed within 15 min. As a control experiment, mice were sacrificed in the dark a ZT14. Brains were collected and SCN tissue isolated. For immunofluorescence experiments, mice were perfused with 4% PFA.

### Cell culture

Mouse embryonic fibroblasts (MEFs) were obtained from mice without functional *Per2* in all body cells^42^, or wild-type mice. Briefly, mice containing the ubiquitously expressed *Cre recombinase* transgene (European Mouse Mutant Archive (EMMA) EM:01149, B6.129-Cre-Deleter: B6.C(129)-Tg(CMV-cre)1Cgn/CgnIbcm) crossed with a conditional *Per2* allele^42^ were backcrossed over ten generations to obtain *Per2* homozygous knock-out mice without *Cre recombinase*. The MEFs subsequently were serially diluted every three days over 30 passages to yield immortalized cells. All lines were maintained in Dulbecco's modified Eagle's medium (DMEM) containing 4.5 g/l glucose, stable glutamine, sodium pyruvate and 3.7 g/l NaHCO_3_ (PAN Biotech), supplemented with 10% fetal calf serum (FCS) and 100 U/mL penicillin–streptomycin at 37 °C in a humidified atmosphere containing 5% CO_2_. Forskolin stimulation (10–100 µM) was used to mimic *in vitro* the molecular pathway activated by light in mice. Samples were collected at specific time points mentioned in the text.

### Plasmids and transfection

The following plasmids were used for the project:

- *ICAP-NLS* Vector carrying the two domains KID (CREB) and KIX (CBP) fused with fluorophores as described before, kindly provided by Prof. Dominique Glauser (University of Fribourg, CH)^48^.
- *Scramble Sh RNA* vector used as a control for the in vitro silencing of *Per2* (Origene cat #TR30021).
- *Sh RNA Per2* Vector used for silencing Per2 in cell lines. (Origene cat #TL501620).

Sequence: 5′-ATGAGCAGTGGCTCCAGCGGAAACGAGAA-3′.

NIH 3T3 cells were co-transfected with either ICAP_NLS/ Scramble shRNA or ICAP_NLPS/shRNA *Per2* in 2 cm dishes at about 70% of their total confluency using linear polyethyleneimine (LINPEI25; Polysciences Europe). The amounts of expression vectors were adjusted to yield comparable levels of the expressed protein. Thirty hours after transfection, cells were induced with forskolin, and the appropriate experiment was performed.

### RNA extraction from cells

Cells were grown to confluency on 6 cm Petri dishes and induced with 10 µM forskolin (50 mM stock in dimethyl sulfoxide) for the indicated time. Total RNA was extracted using the Nucleospin RNA II kit (Macherey & Nagel) and adjusted to 1 µg/µl with water. An amount of 1 µg was reverse-transcribed using Superscript II with random hexamer primers (Thermo Fisher). Real-time PCR was performed using the KAPA probe fast universal master mix and the indicated primers on a Rotorgene 6000 machine. The relative expression was calculated compared to the geometric mean of expression of the inert genes Nono, SirT2, Atp5h, and Gsk3b^62^. For a complete list of primers used in the paper, please see Table 1.

**Table 1:** List of primers used in the paper (5′–3′; FAM: 6-fluorescin, BHQ1: black hole quencher 1).

Name of the primer	Sequence	Use
Per1_ChIP_CREB_FW	*FW*: CAG CCT CCC TGC CCC ACA TT *RV*: GAG AGG GAG GTG ACG TCA AAG C *TM*: FAM-CCA GCT GCC TCG CCC CGC CT-BHQ1	Amplification of a region within the first intron containing a CRE element
Per1_ChIP_control	*FW*: AGG CAC CAG AAA CCT CTT G *RV*: GGC GTA GAT CTG ACA GGC TA *TM*: FAM-TGC CAG AGT CTC CAA AGT ATG CCC AC-BHQ1	Amplification of a region within the promoter
pPer1	*FW*: GGC ATG ATG CTG CTG ACC ACG *RV*: GGT GGG GAT GGG CTC TGT GA *TM*: FAM-TGG CCC TCC CTC ACC TTA GCC TGT TCC T-BHQ1	Precursor of *Per1* mRNA used for the RT-qPCR
pPer2	*FW*: CAC CCA CCC ACC CAC GTG AT *RV*: GGC TGG GAA CTC GCA CTT CCT T *TM*: FAM-CCC TCG TGC AGG TAC CTG GAG AGC -BHQ1	Precursor of *Per2* mRNA used for the RT-qPCR
pcFos	*FW*: GTG AAG ACC GTG TCA GGA GGC A *RV*: CCC AGC CCA CAA AGG TCC AGA A *TM*: FAM-AGC GCA GAG CAT CGG CAG AAG GGG C-BHQ1	Precursor of *cFos* mRNA used for the RT-qPCR
pBmal1	*FW*: GAT CCG AGT GCG GGT GCG *RV*: CGC AGC CAT GCC GAC ACT CA *TM*: FAM- CGG GCG CTC GCA GCG AGC CA-BHQ1	Precursor of *Bmal1* mRNA used for the RT-qPCR
pCry1	*FW*: CTG GTT CGC CGG CTC TTC CA *RV*: GAC ACC CGA CTC GCG CAC A *TM*: FAM- AGG TGG CGG TGA GTC CGA AGC GCT-BHQ1	Precursor of *Cry1* mRNA used for the RT-qPCR
pCry2	*FW*: TGC TAC TGC CCT GTG GGC TT *RV*: GGG AGG CTC CAG AGC CAA AGA *TM*: FAM-CGG CCG ACG TAC AGA CCC CAG TGG-BHQ1	Precursor of *Cry2* mRNA used for the RT-qPCR
pClock	*FW*: TGT AGT GGA GCC GGA AGC A *RV*: GTG CCA AGC CAG GTT CTG A *TM*: FAM-CCC GCC TTG TAG TGG TGC CCA G-BHQ1	Precursor of *Clock* mRNA used for the RT-qPCR
Gsk3β	*FW*: CCA CCT CCT TTG CGG AGA GC *RV*: CTG TGG TTA CCT TGC TGC CAT CT *TM*: FAM-TGC AAG CCA GTG CAG CAG CCT TCA GCT-BHQ1	Probe for *Gsk3β* mRNA used as a normalizer for RT-qPCR
Atp5h	*FW*: TGC CCT GAA GAT TCC TGT GCC T *RV*: ACT CAG CAC AGC TCT TCA CAT CCT *TM*: FAM-TCT CCT CCT GGT CCA CCA GGG CTG TGT-BHQ1	Probe for *Atp5h* mRNA used as a normalizer for RT-qPCR
Sirt2	*FW*: CAG GCC AGA CGG ACC CCT TC *RV*: AGG CCA CGT CCC TGT AAG CC *TM*: FAM- TGA TGG GCC TGG GAG GTG GCA TGG A-BHQ1	Probe for *Sirt2* mRNA used as a normalizer for RT-qPCR
Nono	*FW*: TCT TTT CTC GGG ACG GTG GAG *RV*: GTC TGC CTC GCA GTC CTC ACT *TM*: FAM- CGT GCA GCG TCG CCC ATA CTC CGA GC-BHQ1	Probe for *Nono* mRNA used as a normalizer for RT-qPCR

### RNA extraction from the SCN

RNA from SCN samples was isolated using the Macherey-Nagel RNA Plus kit. Subsequently, 500 ng of purified RNA was used for producing cDNA by reverse transcription (Invitrogen SuperScript II). Real-time PCR was performed using the KAPA probe fast universal master mix and the indicated primers on a Rotorgene 6000 machine.

### Chromatin immunoprecipitation

Chromatin immunoprecipitation from cells was performed as described before^63^. Briefly, the cells were grown to confluency on 15 cm Petri dishes, induced with 10 µM forskolin (50 mM stock in dimethyl sulfoxide), and fixed at the indicated time with 1% formaldehyde/1 × phosphate-buffered saline buffer (PBS) for 10 min at 37 °C. Then the cells were washed twice with ice-cold 1 × PBS and scraped in 1 ml of 10 mM dithiothreitol/100 mM tris(hydroxymethyl)aminomethane (Tris) HCl, pH 8.8. Cells were lysed in 10 mM ethylenediaminetetraacetic acid (EDTA), 1 mM ethyleneglycol-bis(2-aminoethylether)-*N*, *N*, *N*′, *N*′-tetraacetic acid (EGTA), 10 mM 4-(2-hydroxyethyl)-1-piperazineethanesulfonic acid (HEPES), pH 7.6, 0.2% Triton X-100 for 5 min on ice and the obtained nuclei washed with 1 mM EDTA, 0.5 mM EGTA, 200 mM NaCl, 10 mM HEPES, pH 7.6. The purified nuclei were sonicated in 2 mM EDTA, 150 mM NaCl, 20 mM Tris, pH 7.5, 1% SDS using a BRANSON SLPe sonicator with a 4C15 tip for 6 cycles of 10 s each kept on ice, and diluted 1:10 with 2 mM EDTA, 150 mM NaCl, 20 mM Tris, pH 7.5, 1.1% Triton X-100. Equal amounts of chromatin were incubated with the indicated antibodies for 1 h at RT. (Table 2) and the immune complexes were captured with protein A agarose fast-flow beads (Sigma-Aldrich) for 1 h at RT. The beads were washed with 2 mM EDTA, 150 mM, 20 mM Tris, pH 7.5, 0.1% SDS, 1% Triton X-100, then 2 mM EDTA, 500 mM, 20 mM Tris, pH 7.5, 0.1% SDS, 1% Triton X-100, then 2 mM, 250 mM LiCl, 20 mM Tris, pH 7.5, 0.5% Na-deoxycholate, 0.5% NP40 substitute, and finally 2 mM EDTA, 150 mM, 20 mM Tris, pH 7.5. DNA fragments were eluted, and the crosslinks reversed in 2 mM EDTA, 150 mM NaCl, 20 mM Tris, pH 7.5, 1% SDS at 65 °C overnight. The DNA fragments were purified using a MinElute PCR fragment purification kit (Qiagen). Real-time PCR reactions were performed using the KAPA probe fast universal master mix and the indicated primers on a Rotorgene 6’000 machine. The efficiency of the precipitations was calculated by comparing the amount of precipitated material to 1% of the starting material.

**Table 2:** List of antibodies used in the paper and relative applications.

Product name	Catalog reference	Application	Dilution
Rabbit mab Per2	PER21-A Alpha Diagn. (Lot # 869900A1.2-L)	ChIP	1:5
		Western blot	1:1000
		Immunofluorescence	1:200
Rabbit mab CREB	D76D11 cell signaling	ChIP	1:500
		Western blot	1:1000
Rabbit mab CREB (phospho S133)	ab194687 Abcam	ChIP	1:5
		Western blot	1:1000
		Immunofluorescence	1:100
Rabbit mab TORC1 (CRTC1)	ab264144 Abcam	ChIP	1:5
		Western blot	1:1000
Mouse mab Lamin B1	sc-374015 SCBT	Western blot	1:1000
Rabbit mab CBP	PA5-27369 Thermofisher	ChIP	1:5
		Western blot	1:1000
		Immunofluorescence	1:200
Rabbit mab acetyl-histone H3 (Lys27)	D5E4 cell signaling	ChIP	1:5
		Western blot	1:1000
		Immunofluorescence	1:100

### Protein extraction from cells

Total confluent cells plated in 10 cm dishes were washed two times with 1 × PBS (137 mM NaCl, 7.97 mM Na_2_HPO_4_ × 12 H_2_O, 2.68 mM KCl, 1.47 mM KH_2_PO_4_). Then PBS was added again, and plates were kept for 5 min at 37 °C. Cells were detached and collected in tubes and frozen in liquid N_2_. They were subsequently resuspended in Ripa buffer (50 mM Tris-HCl pH7.4, 1% NP-40, 0.5% Na-deoxycholate, 0.1% SDS, 150 mM NaCl, 2 mM EDTA, 50 mM NaF) with freshly added protease or phosphatase inhibitors, and homogenized by using a pellet pestle. Homogenates were kept in ice for 15 min followed by sonication (10 s, 15% amplitude). Right after, the samples were centrifuged for 15 min at 16,100 g at 4 °C. The supernatant was collected in new tubes and pellet discarded.

### Protein extraction from SCN tissue

Isolated SCNs obtained from 5 different mice were pooled together according to the specific condition (either dark or 15 min after the light pulse). The pooled tissues were frozen in liquid N_2_ and resuspended in a brain-specific lysis buffer (50 mM Tris-HCl pH 7.4, 150 mM NaCl, 0.25% SDS, 0.25% sodium deoxycholate, 1 mM EDTA). They were subsequently homogenized using a pellet pestle kept on ice for 30 min and vortexed five times for 30 s each time. The samples were sonicated (10 s, 15% amplitude) and subsequently centrifuged for 20 min at 16,000 g at 4 °C. The supernatant was collected in new tubes and the pellet discarded.

### Immunoprecipitation

Circa 400 µg of protein extract was diluted with the appropriate protein lysis buffer in a final volume of 250 µl and immunoprecipitated using the indicated antibody (ratio 1:50) at 4 °C overnight on a rotary shaker. The day after, samples were captured with 50 µl of 50% (w/v) protein-A agarose beads (Roche) for 3 h at 4 °C on a rotary shaker. Before use, beads were washed three times with the appropriate protein buffer and resuspended in the same buffer (50% w/v). The beads were collected by centrifugation and washed three times with NP-40 buffer (100 mM Tris-HCl pH7.5, 150 mM NaCl, 2 mM EDTA, 0.1% NP-40). After the final wash, beads were resuspended in 2% SDS 10%, glycerol, 63 mM Trish-HCL pH 6.8, and proteins were eluted for 15 min at RT. Laemmli buffer was finally added, samples were boiled for 5 min at 95 °C and stored at – 20 °C.

### Western blot

The indicated amount of protein was loaded onto 10% SDS-PAGE gel and run at 100 Volt for two hours. Once the migration was completed, we performed a semidry transfer (40 mA, 1 h 30 s) using Hybond® ECL™ nitrocellulose membranes followed by red ponceau staining (0,1% of Ponceau S dye and 5% acetic acid) to validate the success of the transfer. The membrane was subsequently washed with TBS 1x/Tween 0.1% and blocked with TBS 1x/Milk 5%/Tween 0.1% for 1 h. After washing, the membrane was stained with the appropriate primary antibodies (Table 2) overnight. The day after, membranes were washed three times with TBS 1x/Tween 0.1% followed by secondary antibody immunoblotting for 1 h at room temperature. The densitometric signal was digitally acquired with the Azure Biosystem.

### Immunofluorescence

Mice were cardiovascularly perfused with 0.9% NaCl followed by 4% PFA and brains were collected. They were subsequently cryoprotected by 30% sucrose solution, and sections of SCN were obtained (30 µm, coronal) using a cryostat. Selected sections were washed three times with 1 × TBS (0.1 M Tris/0.15 M NaCl) followed by 2 × SSC (0.3 M NaCl/0.03 M tri-Na-citrate pH 7). Antigen retrieval was performed with 50% formamide/2 × SSC by heating to 65 °C for 50 min followed by washing 2 × SSC and three times in 1 × TBS pH 7.5. Slices were blocked for 1.5 h in 10% fetal bovine serum (Gibco)/0.1% Triton X-100/1 × TBS at RT.

After the blocking, the primary antibodies (Table 2) were added to the sections and incubated overnight at 4 °C. The next day, sections were washed with 1 × TBS and incubated with the appropriate fluorescent secondary antibodies diluted 1:500 in 1% FBS/0.1% Triton X-100/1 × TBS for 3 h at RT. (Lot: 132876, Alexa Fluor647-AffiniPure Donkey Anti-Mouse IgG (H + L) no. 715–605–150, Lot: 131725, Alexa Fluor647-AffiniPure Donkey Anti-Rabbit IgG (H + L) no. 711–602–152, Lot: 136317 and all from Jackson Immuno Research). Tissue sections were stained with DAPI (1:5000 in PBS; Roche) for 10 min. Finally, the tissue sections were rewashed twice in 1 × TBS and mounted on glass microscope slides. Fluorescent images were taken using a confocal microscope (Leica TCS SP5), and pictures were taken with a magnification of 10 ×, 20 ×, or 63 ×. Images were processed with the Leica Application Suite Advanced Fluorescence 2.7.3.9723, according to the study by^64^.

### Live FRET imaging

Transfected NIH3T3 cells were starved for 4 h with 0.5% FBS DMEM. After starvation (0.5% FBS in DMEM), the medium was removed, and cells were washed twice with 1 × HBTS without CaCl_2_ and MgCl_2_ (25 mM HEPES, 119 mM NaCl, 6gr/L Glucose, 5 mM KCl) gently to avoid cell disturbance. One plate at the time was imaged. Therefore, the medium was changed shortly before the imaging started. NIH3T3 cells were imaged using an inverted epifluorescence microscope (Leica DMI6000B) with an HCX PL Fluotar 5x/0.15 CORR dry objective, a Leica DFC360FX CCD camera (1.4 M pixels, 20 fps), and EL6000 Light Source, and equipped with fast filter wheels for FRET imaging. Excitation filters for CFP and FRET: 427 nm (BP 427/10). Emission filters for CFP: 472 (BP 472/30) and FRET: 542 nm (BP 542/27). Dichroic mirror: RCY 440/520. One frame every 20 s was acquired for at least 90 cycles (0.05 Hz frequency), and the recording lasted at least 30 min. This acquisition rate was ideal to avoid cellular photobleaching and phototoxicity during long recordings. The baseline response in the presence of HBTS only was recorded for 2 min and 40 s. At minute 3:00, 100 µM Forskolin, 2 mM CaCl_2_, and 2 mM MgCl_2_ were added to the cells. To avoid buffer perturbations due to the addition of the drug stimulus, the latter was added between frames, so it had approximately 20 s to diffuse and activate a response in the cells.

### FRET imaging analysis

The time-lapse recordings were analyzed using LAS X software (Leica), adapting a previously described method^65^. Briefly, two regions of interest (ROI) were selected for each cell, and 50 cells per plate were chosen randomly. A first ROI delimiting the background and a second ROI including the cell nucleus of NIH3T3 cell expressing NLS KIDKIX were used per cell. For each channel, the ROIbackground values were subtracted from the ROIcell values. For baseline normalization, the FRET ratio R was expressed as a ΔR/R, where ΔR is R–R0 and R0 is the average of R over the last 120 s prior stimulus.

### Statistical analysis

Statistical analysis of all experiments was performed using GraphPad Prism8 software. Depending on the type of data, either an unpaired t-test with Welch's correction or a paired t-test, when a sample was set to 1, were performed. Two-way ANOVA was performed on time and genotype-dependent experiments. Values considered significantly different are highlighted, *p < 0.05, **p < 0.01, or ***p < 0.001.

## Supplementary Information

Supplementary Figures.
