# Supplementary material for: PER2 mediates CREB-dependent light induction of the clock gene Per1
Source: Sci Rep. 2021 Nov 5;11:21766. doi: 10.1038/s41598-021-01178-6 (PMC8571357; doi:10.1038/s41598-021-01178-6)
Supplement: Supplementary file 1 — Supplementary Figures. [file 41598_2021_1178_MOESM1_ESM.docx]

**SUPPLEMENTARY INFORMATION**

**PER2 mediates CREB-dependent light induction of the clock gene *Per1***

Andrea Brenna^1,2^, Jürgen A. Ripperger^1^, Gabriella Saro^1^, Dominique Glauser^1^, Zhihong Yang^2^, and Urs Albrecht^1*^

^1^ Department of Biology, Faculty of Science and Medicine, University of Fribourg, Fribourg, Switzerland.

^2^ Department of Endocrinology, Metabolism, Cardiovascular System, Laboratory of Cardiovascular and Aging Research, Faculty of Science and Medicine, University of Fribourg, Fribourg, Switzerland.

**Supplementary Figure S1**

**Supplementary Figure S2**

**Supplementary Figure S3**

**Supplementary Figure S4**

**Supplementary Figure S5**

**Supplementary Figure S6**

**Gels and blots**

**Supplemental Figure 1. Induction of *Per1*, *Per2*, *Bmal1*, *Cry*, *Clock* and *cFos* gene expression after light or forskolin stimulation**

(A) Relative pre-mRNA of *Per1* (top panel) and *Per2* (bottom panel) in the SCN of wild type (wt, black bars) and *Per2* knock-out (KO, grey bars) mice after a 15 min light pulse (LP) applied at ZT14. Values are the mean ± SEM. (B) Relative pre-mRNA of *Per1* (top panel) and *Per2* (bottom panel) in immortalized wt or *Per2* KO fibroblast cells after forskolin treatment. Values are the mean ± SD. (C) Relative pre-mRNA of *Bmal1* (left panel) and *Cry1* (right panel) in the SCN of wild type (wt, black bars) and *Per2* KO (grey bars) mice after a 15 min light pulse (LP) applied at ZT14. Values are the mean ± SEM. (D) After forskolin treatment, the relative pre-mRNA of *Bmal1*, *Cry1,* *Clock* and *Cry2* in immortalized wt or *Per2* KO fibroblast cells are shown. Values are the mean ± SD. (E) Relative pre-mRNA of in the SCN of wild type (wt, black bars) and *Per2* KO (grey bars) mice after a 15 min light pulse (LP) applied at ZT14. Values are the mean ± SEM. (F) After forskolin treatment, the relative pre-mRNA of *cFos* in immortalized wt or *Per2* KO fibroblast cells are shown. Values are the mean ± SD. (G) The left panel shows fold change of *Icer* expression in immortalized wt or *Per2* KO fibroblast cells after forskolin treatment. Values are the mean ± SD. Student's t-test with Welch's correction, n = 3, * p < 0.05, ** p < 0.01. The right panel depicts the relative pre-mRNA of *Icer* after forskolin treatment in immortalized wt or *Per2* KO fibroblast cells. Values are the mean ± SD. (H) The left panel shows fold change of *Nor1* expression in immortalized wt or *Per2* KO fibroblast cells after forskolin treatment. Values are the mean ± SD. Student's t-test with Welch's correction, n = 3, * p < 0.05. The right panel depicts the relative pre-mRNA of *Nor1* after forskolin treatment in immortalized wt or *Per2* KO fibroblast cells. Values are the mean ± SD. (I) The left panel shows fold change of *Nur77* expression in immortalized wt or *Per2* KO fibroblast cells after forskolin treatment. Values are the mean ± SD. Student's t-test with Welch's correction, n = 3, * p < 0.05. The right panel depicts the relative pre-mRNA of *Nur77* after forskolin treatment in immortalized wt or *Per2* KO fibroblast cells. Values are the mean ± SD.

**Supplemental Figure 2. Structure of the *Per1* promoter and interaction of PER2 and CREB in cells and the SCN**

(A) Schematic diagram of the *Per1* promoter. The blue circles indicate the regions used as probes for the ChIP assay. The control region contained a Bmal1 binding site. The CRE-element lies in intron 1 1750 bp upstream of the transcription initiation site in exon 2. (B) Top panel: ChIP of BMAL1 on the CRE-element of the *Per1* promoter in wt (black bars) and *Per2* KO (grey bars) fibroblast cell lines after forskolin treatment. Bottom panel: Control ChIP on an unrelated promoter region of *Per1.* Note that 10x more binding of BMAL1 is observed as on the CRE element due to a BMAL1 binding site in this control region. Values are the mean ± SD. (C) IP of CREB pulls down PER2 after forskolin treatment in *wt* cells, but not in *Per2*^-/-^ cells. (D) IP of CREB pulls down PER2 in *wt* SCN extracts but not in *Per2*^-/-^ SCN. * unspecific band recognized by the αPER2 antibody in tissue extracts.

**Supplemental Figure 3. Interaction of CRTC1 with CREB and PER2**

(A) IP of CREB pulls down CRTC1 after forskolin treatment in NIH 3T3 cells. The right panel shows the quantification of IP to input signal. Values are mean ± SD, paired t-test, n=3, * p < 0.05. (B) Control IP experiment in cells with rabbit IgG. CRTC1 and CREB are not precipitated with IgG. An unspecific band is observed using anti-CREB antibody detecting a protein smaller than CREB. (C) IP of CREB in SCN extracts before and after a 15 min light pulse (LP) at ZT14. Co-IP of CRTC1 is only observed after LP. (D) IP of CRTC1 pulls down PER2 in *wt* SCN extracts. kD = kilo Dalton. * unspecific band recognized by the αPER2 antibody in tissue extracts.

**Supplemental Figure 4. Specificity of the PER2 antibody in cells and SCN and CREB phosphorylation after forskolin treatment as evidenced by immunohistochemistry**

(A) The PER2 antibody recognizes PER2 (red) in wt cells but does not detect it in *Per2^-/-^* cells. Blue color = DAPI staining to visualize nuclei. Scale bar = 17 µm. (B) Phosphorylation of CREB at Ser-133 (red) is induced in cells after forskolin treatment, independently of *Per2*. Blue color = DAPI staining to visualize nuclei. Scale bar = 17 µm. (C) The PER2 antibody recognizes PER2 (red) in wt SCN but does not detect it in *Per2^-/-^* SCN. Blue color = DAPI staining to visualize nuclei. Scale bar = 121 µm.

**Supplemental Figure 5. Single cell traces and additional samples**

(A) Single cell traces of control scramble transfected cells (top) and shPer2 transfected cells (bottom) both with ICAP. (B) Additional plate (2 and 3) measurements. The FRET/CFP signal ration changes in response to forskolin treatment in cells transfected with either an scr control (red) or shPer2 (blue) expressing construct. Values are the mean ± SD. Two-way ANOVA revealed a significant difference between the curves, n = 50 (per plate), p < 0.0001.

**Supplemental Figure 6. ChIP of CREB on the *cFos* promoter and example of a Western blot showing Acetylation of H3K27 after a light pulse**

(A) ChIP of CREB on the CRE-element of the *cFos* promoter in wt (black bars) and *Per2* KO (grey bars) fibroblast cell lines after forskolin treatment. Values are the mean ± SD, n = 3.

(B) Western blot experiment of *wt* and *Per2* KO SCN before and after an LP at ZT14. Acetylation of H3K27 is increased after an LP with a tendentially lower increase in *Per2* KO.

**Gels and blots**

Cropping:

Membranes were cut before the hybridization. We need to perform blots on target proteins with sizes covering the entire membrane from 45 KDa (CREB), the smaller protein up to CBP ( over 250 KDA). Therefore, it is impossible to work with large membranes but only with cropped ones, and each portion is stained with a specific antibody.

The acquisition is made using Azure software. The settings are automatic, we can only play with the exposition time, but once the machine gets the saturation, it does not detect anything else anymore. We increased the contrast of those images where we could not detect the border. For some, increasing the contrast helped to make the border appear. For someone others, it was only saturating the signal. However, although you cannot see the borders, the saturation shows that we did not modify the original data when we assembled the figures, and we did not omit any band. This approach is the best we could do.

Oversaturation of blots:

The immunoprecipitation experiments show co-resolution of two different proteins meaning those proteins interact. So, if one protein is much more abundant than another, one of the two signals will be saturated compared to the other as default, but it is the condition if we want to see a clear co-interaction. This saturation is not observable in most of the normal Western blots (input). In these cases the protein accumulation of each target can be detected independently.

**Fig. 2D**

**Input	IP**

**Fig. 2E**

**Fig. 3B**

**Fig. 3C**

**Fig. 3D**

**Fig. 3E**

**Fig. 3F**

**Fig. 3G**

**Fig. 4C**

**Fig. 4E**

**Fig. S2C**

**Fig. S2D**

**Fig. S3A**

**Fig. S3B**

**Fig. S3C**

**Fig. S3D**

**Fig. S6**

IB: H3
